# Experimental and Modelling of Alkali-Activated Mortar Compressive Strength Using Hybrid Support Vector Regression and Genetic Algorithm

**DOI:** 10.3390/ma14113049

**Published:** 2021-06-03

**Authors:** Khaled A. Alawi Al-Sodani, Adeshina Adewale Adewumi, Mohd Azreen Mohd Ariffin, Mohammed Maslehuddin, Mohammad Ismail, Hamza Onoruoiza Salami, Taoreed O. Owolabi, Hatim Dafalla Mohamed

**Affiliations:** 1 Department of Civil Engineering, University of Hafr Al Batin, Hafar Al-Batin 31991, Saudi Arabia; kalsodani@uhb.edu.sa (K.A.A.A.-S.); walasco2010@gmail.com (A.A.A.); 2 School of Civil Engineering, Faculty of Engineering, Universiti Teknologi Malaysia, UTM Johor Bahru 81310, Malaysia; mohdazreen@utm.my (M.A.M.A.); mohammad@utm.my (M.I.); 3 Forensic Engineering Centre, Institute for Smart Infrastructure & Innovation Construction, School of Civil Engineering, Faculty of Engineering, Universiti Teknologi Malaysia, UTM Johor Bahru 81310, Malaysia; 4 Integrated Center for Research on Construction and Building Materials, King Fahd University of Petroleum and Minerals, Dhahran 31261, Saudi Arabia; muddin@kfupm.edu.sa; 5 College of Computer Science and Engineering, University of Hafr Al Batin, Hafar Al-Batin 31991, Saudi Arabia; hamzaosalami@gmail.com; 6 Physics and Electronics Department, Adekunle Ajasin University, Akungba Akoko 341112, Ondo State, Nigeria; 7 Core Research Facilities, King Fahd University of Petroleum and Minerals, Dhahran 31261, Saudi Arabia; dmhatim@kfupm.edu.sa

**Keywords:** compressive strength, natural pozzolan, genetic algorithm, limestone powder, support vector regression

## Abstract

This paper presents the outcome of work conducted to develop models for the prediction of compressive strength (CS) of alkali-activated limestone powder and natural pozzolan mortar (AALNM) using hybrid genetic algorithm (GA) and support vector regression (SVR) algorithm, for the first time. The developed hybrid GA-SVR-CS1, GA-SVR-CS3, and GA-SVR-CS14 models are capable of estimating the one-day, three-day, and 14-day compressive strength, respectively, of AALNM up to 96.64%, 90.84%, and 93.40% degree of accuracy as measured on the basis of correlation coefficient between the measured and estimated values for a set of data that is excluded from training and testing phase of the model development. The developed hybrid GA-SVR-CS28E model estimates the 28-days compressive strength of AALNM using the 14-days strength, it performs better than hybrid GA-SVR-CS28C model, hybrid GA-SVR-CS28B model, hybrid GA-SVR-CS28A model, and hybrid GA-SVR-CS28D model that respectively estimates the 28-day compressive strength using three-day strength, one day-strength, all the descriptors and seven day-strength with performance improvement of 103.51%, 124.47%, 149.94%, and 262.08% on the basis of root mean square error. The outcome of this work will promote the use of environment-friendly concrete with excellent strength and provide effective as well as efficient ways of modeling the compressive strength of concrete.

## 1. Introduction

Concrete is the backbone of our built-environment, especially in urban areas [1]. The construction of infrastructures, such as bridges, roads, dams, tunnels, high-rise buildings, dams, airports, seaports, power plants, seawalls, wastewater plants, freshwater plants and dykes for social and economic benefits consumed roughly 35 billion tons of concrete [2]. This is generally due to its favorable compressive strength, durability, versatility, global availability of the constituent materials, high fire-resistance, and relatively low cost [3]. The world production of Ordinary Portland cement (OPC), the main binding component in concrete was estimated to be 4.6 billion tons in the year 2015 with a projection of four-fold increase by 2050 [4]. However, the OPC calcination process significantly leads to the emission of 5–8% of global CO_2_ into the atmosphere which has greatly contributed to the depletion of earth’s ozone layer [5,6]. Demand-pull by the low carbon-conscious market has gravitated attention to the use of alkali-activated materials (AAM) or geopolymer concrete in recent years as an alternative to OPC [7].

AAM is a system formed by the reaction of soluble alkaline activator and aluminosilicate precursors [8]. AAM is classified into low calcium or geopolymer (fly ash, metakaolin, and natural pozzolans) and high calcium (blast furnace slag) binders. The main products in low binder AAM could be mainly potassium/sodium silicate hydrate with impregnation of alumina (NASH and KASH) within the formation. In high calcium binders, such as blast furnace slag, that are synthesized with a mild alkali, the main product is calcium alumina silicate hydrate (CASH) [9]. AAM has been identified as an eco-efficient and economically-viable alternative for replacing OPC due to its excellent strength, thermal resistance and low permeability [8,10]. AAM is gaining wide acceptance as a component of a sustainable cementitious binder system. It has a wide range of application in precast concrete in which the alkaline activators can be handled appropriately and the curing methods can be controlled and also it can be used for in-situ construction. The world’s largest geopolymer concrete project was carried out by using 40,000 m^3^ of geopolymer concrete for the construction of heavy-duty pavement at the Brisbane west well camp airport in Australia [11]. Additionally, several road projects were constructed using geopolymer by VicRoads state agency in Australia [12]. Furthermore, several applications and standardization of AAM are being done in Russia, Ukraine, South Africa, Netherlands, UK, and the USA, among others [13,14,15]. Many researchers have successfully synthesized alkali-activated mortars and concretes experimentally from natural raw materials, such as natural pozzolan (NP) [9,16,17], agricultural waste materials, such as rice husk ash [18], palm oil fuel ash (POFA) [18,19,20,21,22], or industrial waste, such as silico-manganese slag (SiMn) [23], ground granulated blast furnace slag (GGBFS) [23,24,25], fly ash (FA) [26], silica fume (SF) [26,27], coal bottom ash [28], paper sludge ash [29], and mine tails [8] with aluminosilicate components.

The determination of material properties for the design of civil engineering structures is of great significance to the construction industry. The compressive strength (CS) of AAM is one of the key mechanical properties that dictates its suitability for structural purposes. The CS of alkali-activated mortar (AAMT) is a function of many parameters, such as the chemical composition of the primary or base materials (precursor), sodium hydroxide molarity (NH), curing temperature, sodium silicate to sodium hydroxide (NS/NH) ratio or silica modulus, alkali to binder ratio [NS + NH)/BD], fine aggregate to binder ratio (FA/BD), water to binder ratio (W/BD), and the curing temperature and duration [9,20,30]. The synthesis of AAMT by using the traditional laboratory procedures is laborious, expensive, and time-consuming. This is because the process involves the preparation, curing, and testing of several specimens [31]. Thus, there is a need to develop an alternative procedure that can eliminate the limitations of the traditional methods. This work models the compressive strength of AAMT prepared with natural pozzolan (NP) and limestone powder waste (LSPW) using hybrid support vector regression and genetic algorithm for the first time.

Support vector regression (SVR) is a machine learning algorithm that is capable of relating descriptors to the desired target through pattern acquisition and generation of support vectors [32,33]. It has been extensively applied in many real-life applications due to its unique features, such as strong mathematical background, non-convergence to a local minimum, and excellent predictive strength in the presence of few data-points and descriptive features [34,35,36,37,38,39]. The user-defined parameters in SVR algorithm, such as regularization factor, epsilon, hyper-parameter lambda, kernel option, and kernel function play a significant role when it comes to model performance [40]. These hyper-parameters are evolutionarily optimized in this research work using a genetic algorithm.

Despite the inevitability of concrete in construction industries, the environmental danger of conventional ordinary Portland cement is of serious concern which necessitates urgent attention to alkali-activated materials as the potential alternative to ordinary Portland cement due to their environmental-friendliness, excellent compressive strength, and low permeability. In order to predict the compressive strength (CS) of AAMs without waste of precious time and other valuable resources while the experimental precision is preserved, this present work models the compressive strength of alkaline activated limestone powder and natural pozzolan mortar (AALNM) using hybrid genetic algorithm (GA) and support vector regression (SVR) algorithm, for the first time. The outputs of the developed models are validated using experimental data of the prepared AALNM specimens with different mixture proportions and compositions. The outcome of the modeling and simulation of the compressive strength of AALNM will facilitate the quick estimation of the compressive strength of AALNM system to a high degree of precision, while it saves valuable time and other material resources.

## 2. Description of the Mathematical Background of the Proposed Hybrid Models

This section presents the mathematical formulation of support vector regression as well as genetic algorithm.

### 2.1. Formulation of Support Vector Regression Algorithm

Support vector regression (SVR) is a machine learning algorithm developed by Vapnik [41,42]. It is the regression counterpart of support vector machine, which is used for solving classification problems. SVR has been known to produce good results even when there are few training samples [31,43]. Let {(*x*_1_, *y*_1_), (*x*_2_, *y*_2_), … (*x_n_*, *y_n_*)} be a set of n training samples with which the regression model is to be built, where *x_i_* ∈ ℜ*^d^* and *y_i_* ∈ ℜ are the set of the input features and the target for the i-th training sample, respectively. The SVR algorithm seeks to find a function *f*(*x*) which fits all the training data with a deviation of at most ε while being as flat as possible [44]. Without loss of generality, *f* can be represented as a linear function as shown in Equation (1) where 〈…〉 denotes the dot product in ℜ*^d^*.

(1) $$f\left(x\right)=\langle w,\;x\rangle +b\;w\;\in {\mathbb{R}}^{d},\;b\;\in \;\mathbb{R}$$

In order to make *f*(*x*) flat, *w* needs to be as small as possible. *w* can be made small by minimizing its Euclidean norm ${||w||}^{2}=\langle w,w\rangle$. The corresponding convex optimization problem can be stated as shown in Equation (2) [45].

(2) $$\mathrm{minimize}\;\frac{{||w||}^{2}}{2}\;\mathrm{subject}\;\mathrm{to}\;\{\begin{array}{c} y_{i}-\langle w,\;x\rangle -b\leq \varepsilon \\ \langle w,\;x\rangle +b-y_{i}\leq \varepsilon \end{array}\;$$

Since Equation (2) may be infeasible, slack variables *ξ_i_* and ξ_i_* are introduced to the optimization problem, yielding Equation (3).

(3) $$\mathrm{minimize}\;\frac{{||w||}^{2}}{2}+C\overset{n}{\underset{i=1}{\sum }}\left(\xi_{i}+\xi_{i}{}^{*}\right)\;\mathrm{subject}\;\mathrm{to}\;\{\begin{array}{c} y_{i}-\langle w,\;x\rangle -b\leq \varepsilon +\xi_{i}\\ \langle w,\;x\rangle +b-y_{i}\leq \varepsilon +\xi_{i}{}^{*}\\ \xi_{i},\;\xi_{i}{}^{*}\geq 0 \end{array}\;$$

The regularization constant *C* controls the trade-off between generalization ability and accuracy on the training set [46]. Equation (3) can be solved more conveniently in its dual form by constructing a Lagrange function from the constraints and objective function, and introducing dual variables [44]. The solution to the dual problem is given in Equation (4):

(4) $$f\left(x\right)=\overset{n}{\underset{i=1}{\sum }}\left(\alpha_{i}+\alpha_{i}{}^{*}\right)\;\phi \langle x_{i},x\rangle +b$$

where *α_i_* and α_i_* are Lagrange multipliers. *φ* is the kernel function that allows the input features to be mapped to higher dimensional feature space. This mapping improves the accuracy and robustness of SVR [47]. In this work, the kernel function that performs better is the Gaussian kernel function and is defined in Equation (5).

(5) $$\phi \langle x_{i},x\rangle =\exp\left(-\frac{||x-x_{i}||}{\sigma }\right)$$

where *σ* stands for the kernel option. Since the performance of SVR depends on the choice of hyper-parameters which include the regularization factor (*C*), epsilon (*ε*), and kernel option (*σ*), genetic algorithm is hybridized with SVR for optimal selection of these parameters.

### 2.2. Description of Genetic Algorithm

Genetic algorithm (GA) is a population-based optimization algorithm inspired by evolution and natural selection, which is capable of solving complex search problems [48]. In GA, potential solutions to a problem are encoded as a chromosome, which is made up of a number of genes. At the start of GA, a population of chromosomes is randomly generated. The fitness level of each chromosome is determined by means of a fitness/cost function. High-quality chromosomes have a greater chance of being selected for reproduction than low-quality chromosomes. During reproduction (crossover), portions of two or more existing chromosomes are combined together to generate one or more offspring. Some chromosomes may undergo mutation, during which small random changes are introduced in a chromosome to prevent it from being stuck in a local optimum [49]. GA goes through several iterations (generations) of reproduction, crossover, and mutation until a stopping condition is met [45].

### 2.3. Acquisition of Experimental Data and Computational Hybridization of the Proposed Models

This section discusses the computational method adopted for hybridization of the two algorithms. The acquisition and description of the dataset used for modelling and simulation are also presented.

#### 2.3.1. Experimental Description and Data Acquisition

Natural pozzolan (NP) was obtained from Imerys Minerals Arabia, Rabigh, Kingdom of Saudi Arabia and the limestone powder waste (LSPW) was collected from the local tile manufacturing plants. Subsequently, it was oven-dried at 105 ± 5 °C for 24 h to remove the moisture. The LSPW was sieved through a 200 µm sieve to remove stones and debris. The alkaline activator used in this study is a combination of commercially available aqueous sodium silicate (SS) with its initial silica modulus (Ms = SiO_2_/Na_2_O) of 3.3 and nM NaOH_(aq)_ (NH) where n is the value of the molarity (4, 6, 8, 10, 12, and 14). The percentage composition of the Na_2_SiO_3(aq)_ are as follows: H_2_O: 62.11%, SiO_2_: 29.13%, and Na_2_O: 8.76%. Desert sand [50] was used as fine aggregate (FA). The fineness modulus of FA was 1.82 and the specific gravity in saturated surface dry (SSD) condition was 2.63 and the water absorption was 0.5%. The mixture proportions of AAMT were designed with the LSPW content of 0%, 20%, 40%, 60%, 80%, and 100% and natural pozzolan contents of 100%, 80%, 60%, 40%, 20%, and 0%, respectively. The samples were designated as AANL_x_ (alkali-activated NP/LSPW mortar), where *x* is the $\frac{L}{L+N}$ ratios. A total of six AANL_x_ (where *x* = 0, 0.2, 0.4, 0.6, 0.8 and 1) mixtures were prepared. The fine aggregate to the binder (FA/B) ratio ranged from 1.4 to 2.2 at an interval of 0.2. The Na_2_SiO_3(aq)_/NaOH_aq_ (NS/NH) ratio ranges from 0 to 1.5 and alkaline activator to binder ratio of 0.45, 0.5 and 0.55 were used. A free water to precursor (pozzolanic material) ratio of 0.1 was used in all the mixtures. The required quantities of constituent materials were measured and mixed in batches in the 5.0 L capacity Hobart planetary bench mixer (Hobart GmbH, Offenburg, Germany). The mixing of the materials was done in two stages. In the initial stage, the NP and LSPW powder and sand were mixed in a dry condition for 3 min. In the second stage, the alkaline solution (NaOH_(aq)_ + Na_2_SiO_3(aq)_) and water were added for the wet mixing stage which involves low speed mixing for 2 min and another 4 min for fast or high speed mixing until a homogeneous mixture was achieved, the total mixing time was about 9 to 10 min to ensure the homogeneity of the mix. Thereafter, the mortar was placed in oil-smeared steel molds measuring 50 × 50 × 50 mm in two layers and each layer was vibrated on the vibrating table for 30 s to remove any entrapped air from the mixture. Then the surface was carefully smoothened with a trowel to have a smooth finish. After placement, consolidation, and finishing of the mortar, the specimens were covered with a plastic sheet to prevent moisture loss and kept in the laboratory at 20 ± 5 °C for 24 h. After 24 h of casting, the cubes were de-molded and placed in zip plastic bags to avoid evaporation of moisture. The specimens were then subjected to temperature curing in an oven maintained at the room temperature (20 ± 5 °C) and various curing temperature of 45, 60, 75, and 90 °C for 24 h. After that, the specimens were cured under a normal room temperature condition of 20 ± 5 °C until the age of testing (1, 3, 7, 14, and 28 days). The CS of the AANL mortar was determined in accordance with ASTM C 39 [51] on 50 × 50 × 50 mm cube specimens using a digital compression testing machine. The CS of the specimens was determined after 1, 3, 7, 14, and 28 days of curing. Three specimens were tested at each age and the average compressive strength values were recorded for use in the models. Table 1 summarizes the quantities of materials and the compressive strength of 390 alkali-activated specimens.

Statistical analysis was carried out on the dataset presented in Table 1 and the outcomes of the analysis are presented in Table 2. The mean and the range presented in the Table 2 give significant insights to the overall content of the dataset while the consistencies in the dataset from one measurement to another is contained in the acceptable standard deviation. The correlation coefficient (CC) shows the degree of a linear relationship between each of the descriptors and the targets. From these coefficients, the inadequacy of linear models to holistically capture the relationship between the descriptors and the targets can be inferred. Hence, the need for nonlinear modelling, such as the proposed hybrid GA-SVR, becomes paramount.

#### 2.3.2. Computational Hybridization of Genetic Algorithm and Support Vector Regression

Due to the fact that the performance of SVR is highly dependent on the set of SVR parameters used, it is important to fine-tune the parameters of SVR in order to obtain a good prediction. All the modelling and simulation were conducted within the MATLAB (2019 MathWorks, Natick, MA, USA) computing environment. Before the commencement of modelling, the dataset was divided into training, testing, and validation in the ratio 6:2:2 after randomization. Randomization of data is necessary to ensure an even distribution of data-points and to promote efficient computation. Therefore, 16 data-points were used for training the model, five data-points were used for tuning the hyper-parameters, while the remaining five data-points were used for validating the robustness of the developed hybrid model. The procedures for hybridization of SVR with GA are itemized below:

Step I: Chromosome representation and population initialization: Each chromosome is real-coded and made up of three genes representing regularization factor, epsilon, and kernel option for the selected kernel function. The search space which represents the range of valid values for each of the parameters is defined as 1–1000 for regularization factor, 0.1–1 for epsilon, and 0.001 to 1 for kernel option. These wide ranges of search space enhance the possibility of attaining the optimality. The initial population of chromosomes was formed by randomly assigning values for each parameter within its lower and upper bounds.

Step II: Fitness value computation: In order to compute the fitness value for each of the chromosome, the kernel function was selected from a pool of functions which include Gaussian, polynomial and sigmoid among others while the hyper-parameter that defines the hyper-plane was set at 10^−7^. Thereafter, the values of other SVR parameters (which include regularization constant, epsilon and kernel option) are encoded in the chromosome. The set of parameters as well as the training set of data were used to train SVR algorithm. The generalization capability and robustness of the SVR prediction model is measured by implementing the model to estimate the compressive strength of data samples contained in the validation set and computing the root mean square error (RMSE) between the estimated compressive strength and the empirically measured comprehensive strength. Therefore, the fitness value of a chromosome is the RMSE of the corresponding SVR model on the validation set of data.

Step III: Selection for reproduction: Based on the principle of survival of the fittest, the chromosomes having higher quality (i.e., lower RMSE) have a greater chance of being selected for reproduction. Tournament selection was utilized in the present hybrid GA-SVR model. In tournament selection, a few chromosomes were randomly chosen from the entire population and the winner of the tournament, which is the fittest of the chosen chromosomes, was used for crossover. The selection probability was set at 0.8.

Step IV: *Crossover:* The crossover operator combines portions of two parents to form two offspring. Subsequences and portions of both parents are swapped among the children with a crossover probability of 0.65.

Step IV: *Mutation:* With a mutation probability of 0.009, the value at a randomly chosen position in a chromosome was altered.

**Figure 1:**
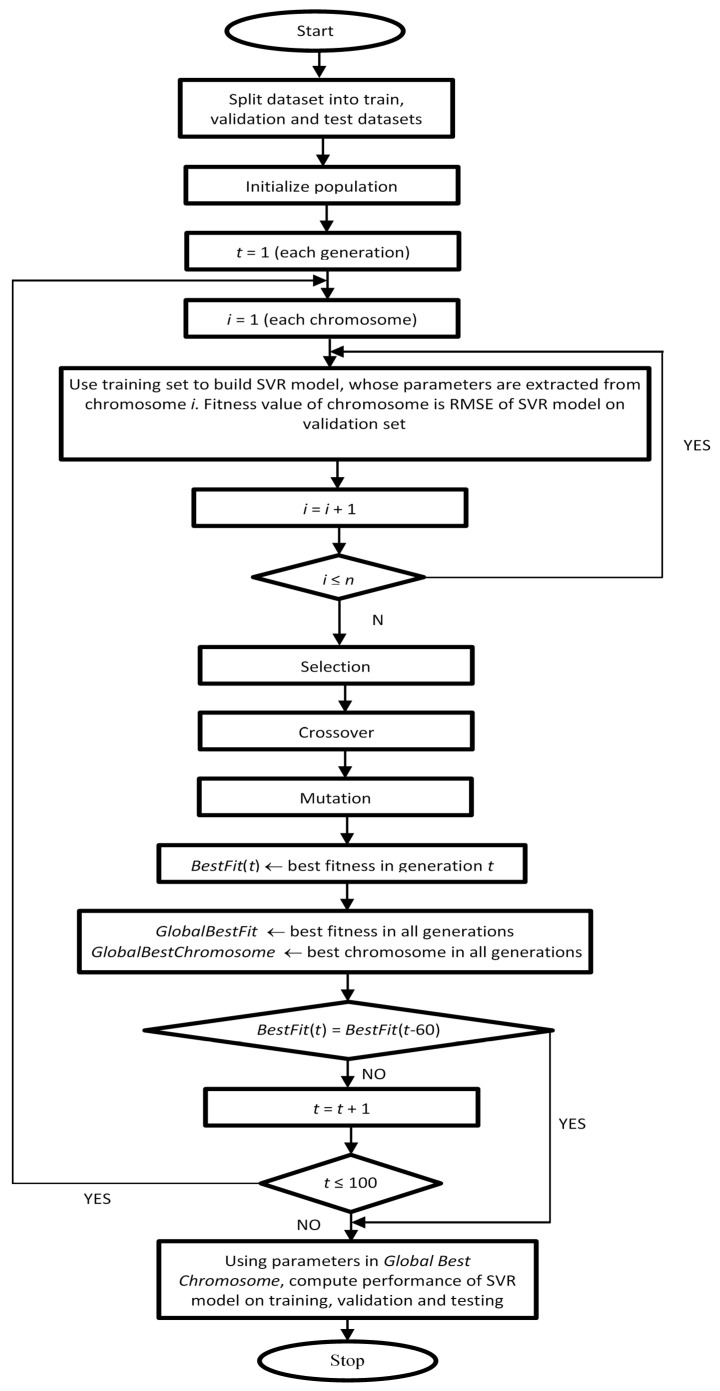
Computational Flow chart of GA-SVR for prediction of compressive strength.

Step V: *Stopping criteria:* Step I to Step IV was repeated until the best fitness value does not improve over 60 consecutive generations. Figure 1 presents the computational flow chat of the hybridization.

## 3. Results and Discussion

This section presents and discusses the results of the modelling, simulation, and optimization. The outcomes of the developed models are compared with the experimental values while the developed models are implemented for modelling the behavior of the compressive strength under various conditions.

### 3.1. Early Age Compressive Strength Using the Developed Hybrid Models

The results of early age compressive strength as estimated using the developed hybrid GA-SVR-CS1, GA-SVR-CS3, GA-SVR-CS7, and GA-SVR-CS14 model for one-, three-, seven-, and 14-days, respectively, are presented in this section. The convergence of the models at various numbers of initial populations is also discussed. The effectiveness of each of the models in obtaining maximum desired compressive strength is also discussed.

#### 3.1.1. Optimization of SVR Hyper-Parameters for the Early and 14-Day Compressive Strength Models

**Figure 2:**
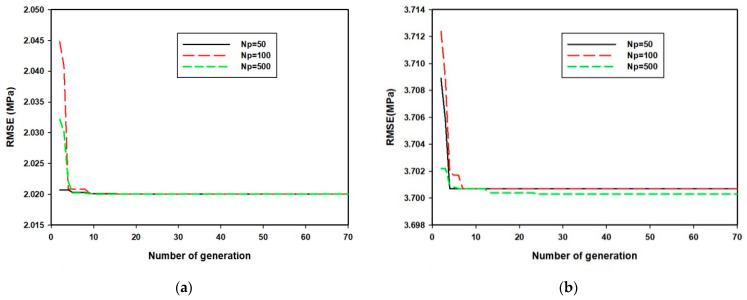
(**a**) Convergence of hybrid GA-SVR-CS1 to the number of generations at different population size (**b**) Convergence of hybrid GA-SVR-CS3 to the number of generations at different population size.

Since the hyper-parameters of SVR algorithm are of high significance to the performance of the model, these parameters were optimized using GA. The convergence of the hybrid GA-SVR-CS1 and GA-SVR-CS3 models is presented in Figure 2.

Figure 2a shows the convergence of GA-SVR-CS1 at different number of initial population while Figure 2b presents the convergence of GA-SVR-CS3 model at three different numbers of initial populations. Both graphs in Figure 2 strongly indicate the robustness of the models as they converge to the same point, despite the large size of the population. Similar convergence is obtained for GA-SVR-CS7 and GA-SVR-CS14 models, developed for estimating seven- and fourteen-days compressive strength, respectively. Only the convergence of GA-SVR-CS1 and GA-SVR-CS3 models is presented so as to prevent repetition. Table 3 presents the obtained hyper-parameters for each of the developed early-age compressive strength models.

#### 3.1.2. Performance of the Developed Early-Age Compressive Strength Models at Each Developmental Stage

The performance of each of the developed models for estimating the strength of AALNM is assessed using correlation coefficient (CC) between the estimated and experimentally measured values, root mean square error (RMSE), and mean absolute error (MAE) for training, testing, and validating the set of data. The values of each of the parameters at each of the developmental stages of the model are presented in Table 4. The validation stage is the most vital stage by which the accuracy as well the generalization and predictive strength of the models can be assessed since the models are subjected to data that are not involved in both training and testing stages. In the training phase, support vectors are acquired while these support vectors are altered and perfected to give excellent estimation using the testing set of data for parameters tuning. The developed GA-SVR-CS1 model performs better at validation stage than training and testing stage with the performance improvement of 17.48% and 47.52%, respectively, using RMSE as a performance measuring parameter. However, a comparison of validation stage with training and testing stage on the basis of MAE shows that the developed GA-SVR-CS1 model performs better during the validation than training and testing stage with performance enhancement of 8.15% and 75.04%, respectively. On the basis of CC, the performance at the validation stage is better than that of the training phase while the best performance is obtained during the testing phase. Table 4 presents the value of each of the performance measuring parameters for each of the early-age compressive strength models. In the case of developed hybrid GA-SVR-CS3 model for estimating three-days compressive strength, the model performs better during the validation stage compared with the training stage while the best performance was recorded during the initial acquisition of support vectors using the three performance measuring parameters as a yardstick for comparison. The testing phase of GA-SVR-CS7 model shows the best performance metrics while in the case of GA-SVR-CS14 model, the training phase of the model demonstrates the best performance.

#### 3.1.3. Comparison between the Experimentally-Measured Early and 14-Day Compressive Strength and the Outcomes of the Developed Hybrid Models

**Figure 3:**
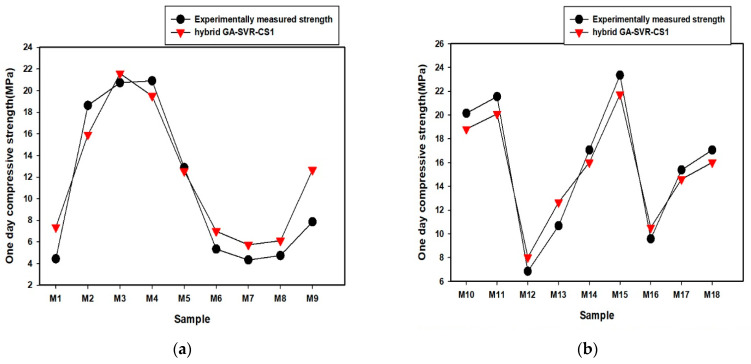

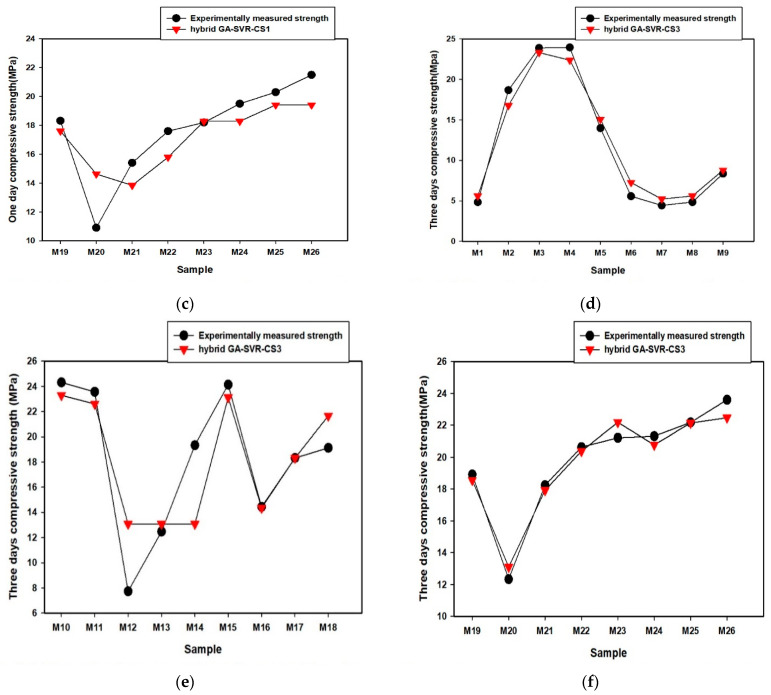
(**a**) Comparison between experimentally measured one-day compressive and results of the developed hybrid GA-SVR-CS1 for samples M1-M9 (**b**) Comparison between experimentally measured one-day compressive and results of the developed hybrid GA-SVR-CS1 for samples M10-M18 (**c**) Comparison between experimentally measured one-day compressive and results of the developed hybrid GA-SVR-CS1 for samples M19-M26 (**d**) Comparison between experimentally measured three-day compressive and results of the developed hybrid GA-SVR-CS1 for samples M1-M9 (**e**) Comparison between experimentally measured three-day compressive and results of the developed hybrid GA-SVR-CS1 for samples M10-M18 (**f**) Comparison between experimentally measured three-day compressive and results of the developed hybrid GA-SVR-CS1 for samples M19-M26.

A comparison of the predicted early-age compressive strength and the measured values for AALNM mixtures is presented in Figure 3 for the developed GA-SVR-CS1 and GA-SVR-CS3 models. The predicted compressive strength is very close to the experimental values, as shown in Figure 3. Similar closeness is obtained for the other two early-age compressive strength models (GA-SVR-CS7 and GA-SVR-CS14). The actual values of the estimated compressive strength for each of the developed early-age compressive strength models are provided in Appendix A.

### 3.2. Modelling the Influence of Constituents of AALNM on the Early-Age and 14-Day Compressive Strength Using the Developed Hybrid Models

**Figure 4:**
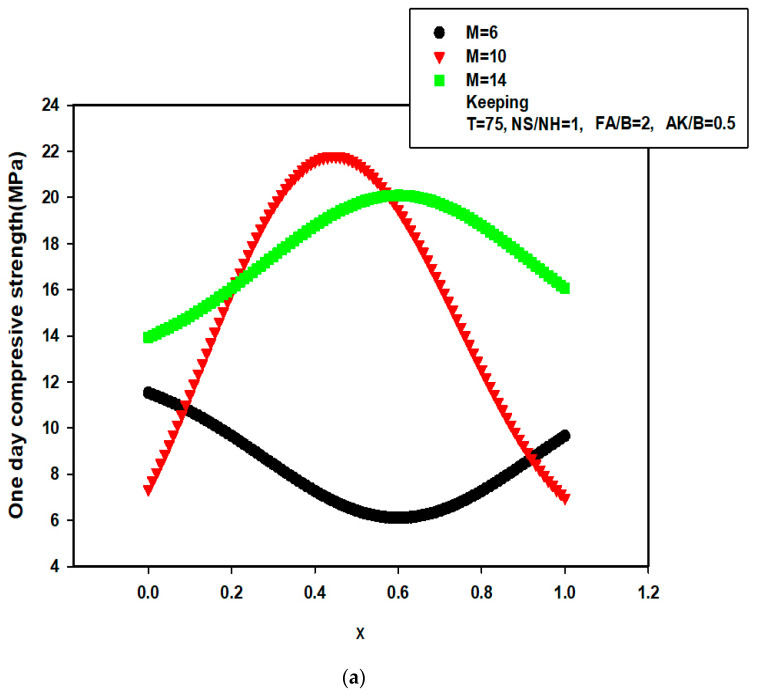

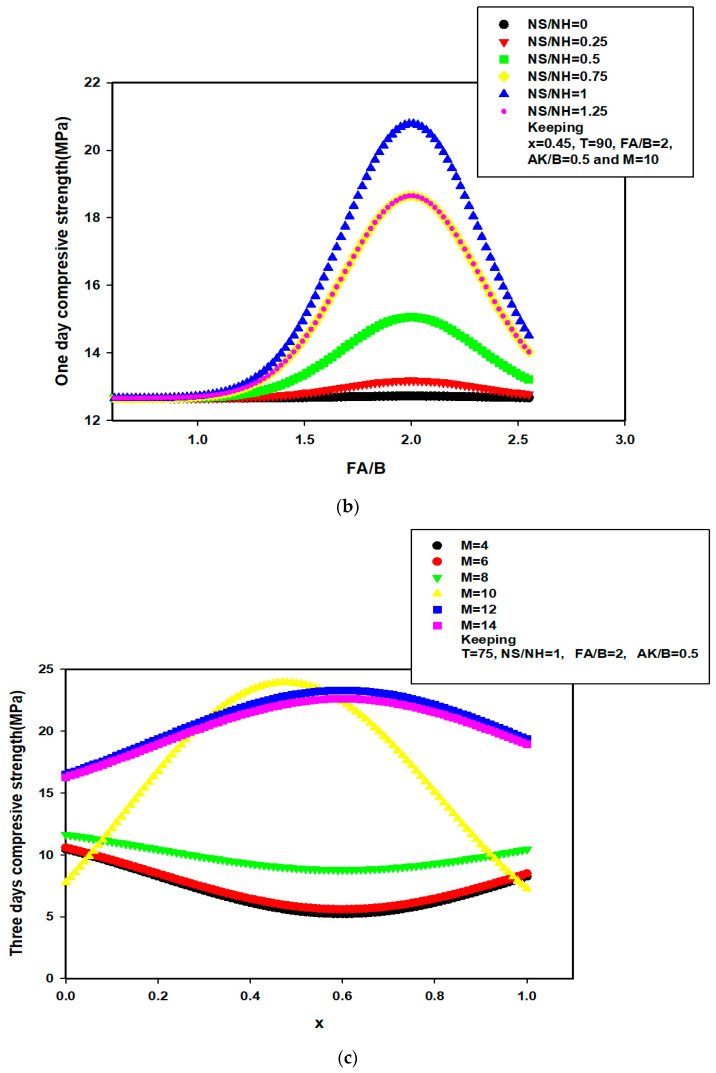

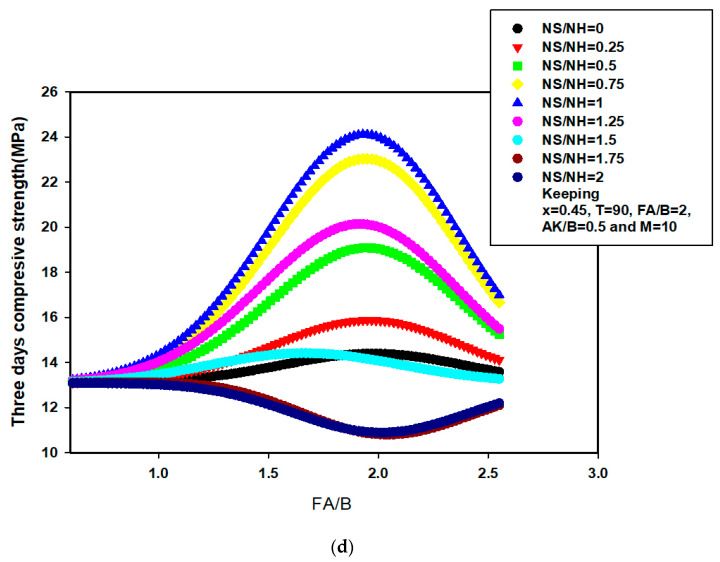
(**a**) Dependence of one day compressive strength of AANLM at various molarity on the ratio of LSP/NP+LSPW using the developed hybrid GA-SVR-CS1 (**b**) Dependence of one day compressive strength of AANLM at various NS/NH on the ratio of FA/B using the developed hybrid GA-SVR-CS1 (**c**) Dependence of three day compressive strength of AANLM at various molarity on the ratio of LSP/NP+LSPW using the developed hybrid GA-SVR-CS3 model (**d**) Dependence of three day compressive strength of AANLM at various NS/NH ratio on the ratio of FA/B using the developed hybrid GA-SVR-CS3 model.

The developed early-age compressive strength models, specifically GA-SVR-CS1 and GA-SVR-CS3, are implemented for investigating the influence of binary binder ratio and the fine aggregate to binder ratio on the early-age compressive strength. This application becomes necessary since the proposed early-age compressive strength models have been trained, tested, and validated with the experimental data and the performance measuring parameters recorded are excellent. Figure 4a shows the dependence of one-day compressive strength on the binary binder ratio (that is, the ratio of limestone powder to natural pozzolan represented as x) at various molarity (represented by M) of sodium hydroxide while keeping the curing temperature (T), sodium silicate to sodium hydroxide (NS/NH) ratio, fine aggregate to binder ratio (FA/B), and alkaline to binder ratio (AK/B) at 75 °C, 1, 2, and 0.5, respectively. It was noted that the one-day compressive strength of AALNM mixture increases with binary binder ratio (except when the molarity of sodium hydroxide is 6), attains maximum strength, and begins to decrease. The one-day compressive strength attains maximum value when the molarity of sodium hydroxide is 10 and the ratio of binary binder ratio is 0.45 [52]. It should be noted here that the developed GA-SVR-CS1 model was only fed with input descriptors while the developed model utilizes its acquired support vectors during training phase to predict the behavior of AALNM mixture under the selected conditions. Figure 4b presents the influence of fine aggregate to binder ratio on one-day compressive strength of AALNM system at various sodium silicate to sodium hydroxide ratios, keeping binary binder ratio at its optimum value of 0.45.

The data in Figure 4b indicates that the maximum one-day strength can only be achieved when FA/B is 2 while NS/NH is kept at 1. The results of similar investigation are presented in Figure 4c,d for three-days compressive strength using the developed GA-SVR-CS3 model. Figure 4c presents the dependence of three-days compressive strength on the binary binder ratio at different molarity of sodium hydroxide while setting the curing temperature (T), sodium silicate to sodium hydroxide (NS/NH) ratio, fine aggregate to binder ratio (FA/B) and alkaline to binder ratio (AK/B) at 75 °C, 1, 2, and 0.5, respectively. The behavior of AALNM mixture is parabolic in nature while the strength at molarity of 4 and 6 show minimum points correspond to minimum possible compressive strength. The results of modeling and simulation as presented in Figure 4c show that the maximum three-days strength possible could be obtained when the binary binder ratio is set at 0.45 while the molarity of sodium hydroxide is maintained at 10. At this optimum binary binder ratio, the significance of FA/B on three-days compressive strength at various values of NS/NH ratio is investigated and the results are presented in Figure 4d. It was observed that after a maximum possible three-days strength at NS/NH ratio of 1, further increase in NS/NH lowers the strength.

### 3.3. Estimation of 28-Days Compressive Strength Using the Developed Hybrid GA-SVR-CS28 Model

This research work also developed models that can effectively estimate the 28-days compressive strength for structural and construction purposes. In this case, five different models were developed with distinct features, capacities and estimation accuracy. The developed hybrid GA-SVR-CS28A model estimates the 28-days compressive strength of AALNM mixture using binary binder variation, sodium hydroxide molarity (NH), curing temperature, sodium silicate to sodium hydroxide (NS/NH) ratio, alkaline to binder ratio (AK/B), and fine aggregate to binder (FA/BD) ratio as input descriptors to the model. Furthermore, the developed hybrid GA-SVR-CS28B and GA-SVR-CS28C models estimate the 28-day compressive strength using one-day and three-day compressive strength as input descriptor while the seven-days compressive strength serves as the descriptor to GA-SVR-CS28D model. Another developed hybrid GA-SVR-CS28E model predicts the 28-day strength using 14-day strength as descriptor. Each of these models have unique importance and significance. The developed hybrid GA-SVR-CS28B model allows engineers and practitioners to predict 28-day compressive strength using the results of one-day compressive strength so as to save precious time and other valuable resources. Since it saves time and preserves experimental precision, the model can be deployed for making quick and precise decision in building industries. Similar advantages are attached to other models, such as GA-SVR-CS28C, GA-SVR-CS28D, and GA-SVR-CS28E. The choice from these models can be decided on the basis of their accuracies and the urgency of the application. It is worth noting that mixtures M10, M11, M17, and M25 exhibited a slight reduction in compressive strength after 14-days. Mixtures M10, M11, M17, and M25 were all synthesized with high concentration of sodium hydroxide NaOH_(aq)_, at higher concentration of NaOH_(aq)_, the early-days compressive strength values are higher due to the rapid geopolymerization process caused by high dissolution rate of monomers in the binder. However, at later stage, excessive presence of OH^−^ in high molar concentration of NaOH_(aq)_ could cause ionic congestion that might prevent the polymerization process. This could limit the formation of C-A-S-H in the formed gels leading to a reduction in the compressive strength as observed in mixtures M10, M11, M17, and M25 [52].

#### 3.3.1. Optimization of SVR Hyper-Parameters for the Developed GA-SVR-CS28 Model

**Figure 5:**
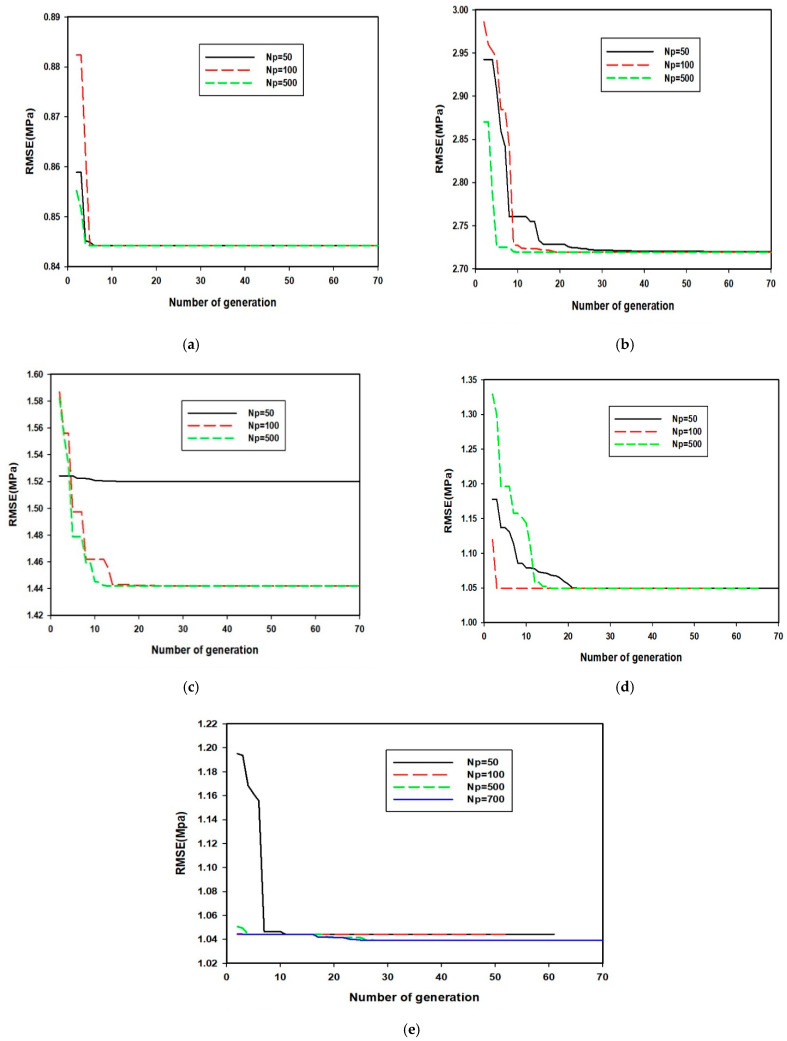
(**a**) Convergence of hybrid GA-SVR-CS28A to the number of generation at different pollution size (**b**) Convergence of hybrid GA-SVR-CS28B to the number of generation at different pollution size (**c**) Convergence of hybrid GA-SVR-CS28C to the number of generation at different pollution size (**d**) Convergence of hybrid GA-SVR-CS28D to the number of generation at different pollution size (**e**) Convergence of hybrid GA-SVR-CS28E to the number of generation at different pollution size.

The hyper-parameters of each of the developed 28-days compressive strength models are optimized using GA. The optimum values of the hyper-parameters are presented in Table 5, while results showing the convergence of the optimization are presented in Figure 5a–e. As shown in Figure 5c, the developed model attained local minimum when fifty of the population were contained within the search space because the space is limited, while the exploration capacity of the model becomes inefficient. An increase in the number of probable solutions in the search space to one hundred strengthens the exploration capacity of the model and the optimum convergence was observed. Among the merits of the implemented GA is that it avoids local minimum convergence, especially when the initial population is varied.

#### 3.3.2. Performance of Hybrid GA-SVR-CS28 at Each Developmental Stage

**Figure 6:**
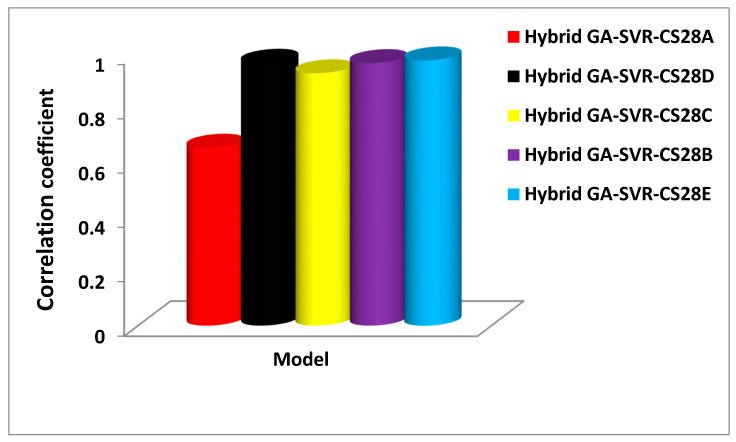
Performance comparison of the developed 28-day compressive strength model using the correlation coefficient performance metric.

The generalization and predictive strength of each of the 28-days compressive strength model are assessed using CC, RMSE, and MAE between the estimated and experimentally measured values for validation set of data. Figure 6 presents the comparison on the basis of correlation coefficient between the estimated strength and the measured values for the validation set of data. The developed hybrid GA-SVR-CS28A model that estimates the compressive strength of AALNM using six input descriptors which include binary binder variation (*x*), sodium hydroxide molarity (NH), curing temperature, sodium silicate to sodium hydroxide (NS/NH) ratio, alkaline to binder ratio (AK/B), and fine aggregate to binder (FA/BD) ratio, shows least performance as characterized with lowest value of CC. The significance of utilizing early-age strength and three-day strength to predict 28-day strength can be observed from the results of GA-SVR-CS28B and GA-SVR-CS28C as both models predict the 28-day strength with 96.64% and 92.88% accuracy, respectively. This significantly saves time in predicting the 28-days compressive strength, utilizing the early-age strength.

**Figure 7:**
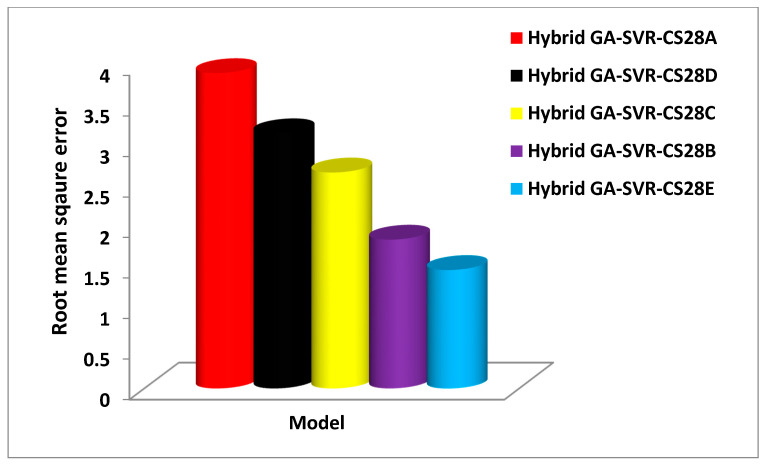
Performance comparison of the developed 28-day compressive strength model using the root mean square error performance metric.

**Figure 8:**
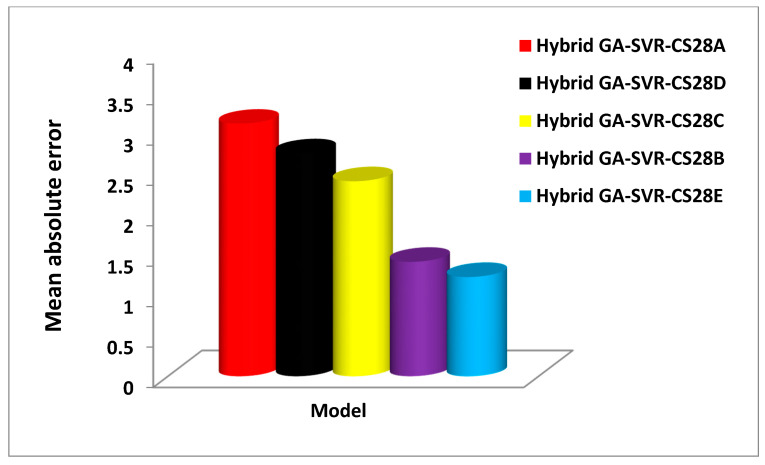
Performance comparison of the developed 28-day compressive strength model using the mean absolute performance metric.

However, hybrid GA-SVR-CS28B model that utilizes one-day strength performs better than GA-SVR-CS28C model that estimates 28-day strength using the three-day compressive strength with improvement of 3.89%. Similarly, the developed hybrid GA-SVR-CS28C model that estimates 28-day strength using seven-day strength as input does this task with an accuracy of 96.75% while the hybrid GA-SVR-CS28D model that uses fourteen-days strength for its 28-day estimation is characterized with an accuracy of 97.6%. Despite the high accuracy that characterizes each of these models, the developed hybrid GA-SVR-CS28C model that estimates 28-day strength using 14-day-strength shows the best performance. Figure 7 compares each of the developed 28-day compressive strength models on the basis of RMSE, while Figure 8 compares the model using MAE as a performance measuring parameter.

The developed hybrid GA-SVR-CS28A model also shows the least performance with the highest RMSE and MAE as respectively presented in Figure 7 and Figure 8. The developed hybrid GA-SVR-CS28B and GA-SVR-CS28C models estimate the 28-day compressive strength with very low RMSE of 1.8432 MPa and 2.669 MPa while MAE characterizing the models is 1.4143 MPa and 2.4128 MPa. In the same vein, the RMSE for implementing the hybrid GA-SVR-CS28D and GA-SVR-CS28E models on validation set of data is 3.1514 MPa and 1.4688 MPa, respectively, while the respective values of MAE are 2.758.83 MPa and 1.226 MPa. On the basis of the recorded errors, the developed hybrid GA-SVR-CS28B model performs better than model GA-SVR-CS28C, while the hybrid model GA-SVR-CS28E shows the best performance. The error values and the coefficient of correlation for each of the developed 28-days compressive strength models are presented in Table 6.

### 3.4. Comparison between the Experimentally Measured 28-Day Compressive Strength and the Outcomes of the Developed Hybrid GA-SVR-CS28 Model

**Figure 9:**
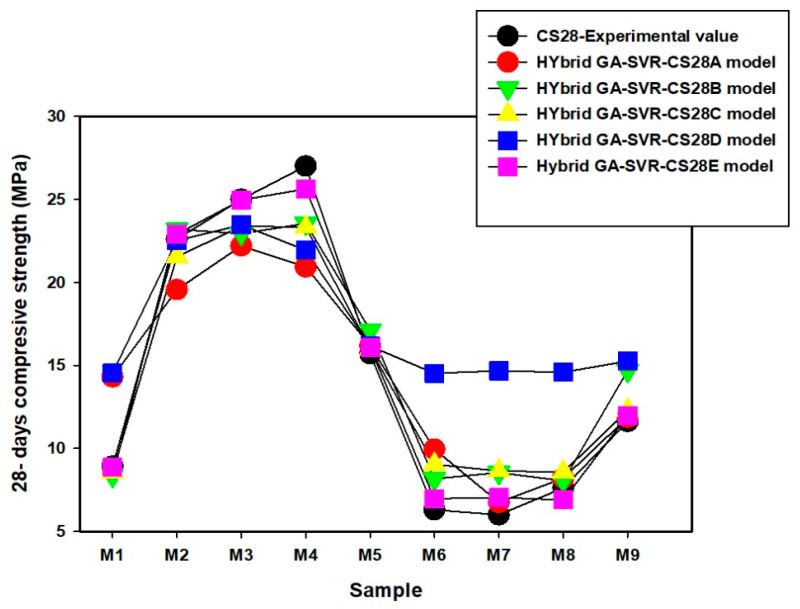
Comparison between experimentally measured 28-day compressive strength and the results of the developed models for mixtures M1 to M9.

**Figure 10:**
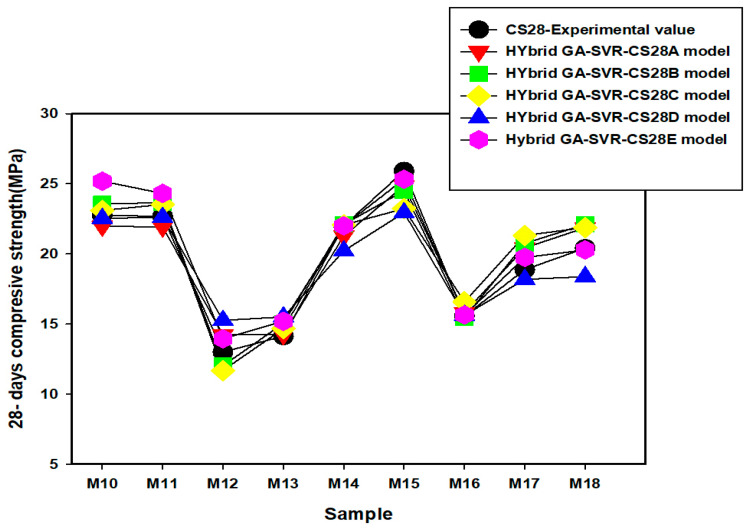
Comparison between experimentally measured 28-day compressive strength and the results of the developed models for mixtures M10 to M18.

**Figure 11:**
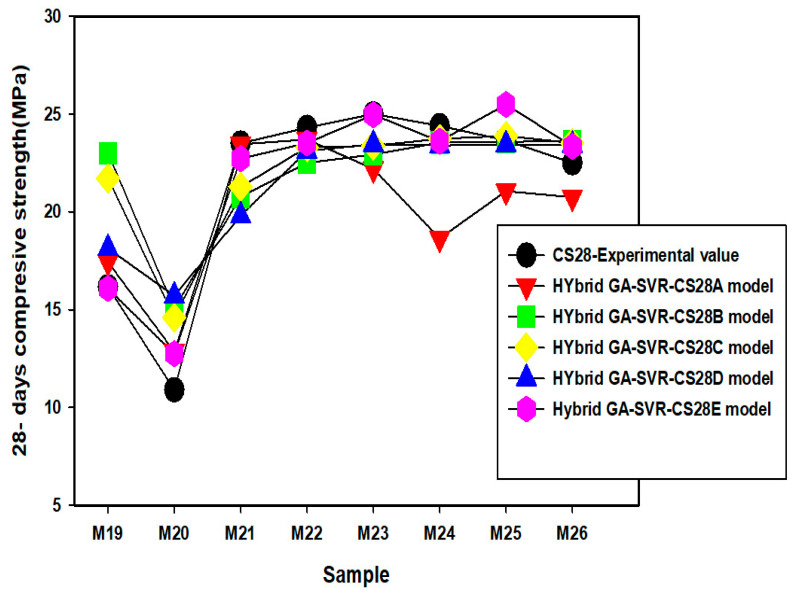
Comparison between experimentally measured 28-days compressive strength and the results of the developed models for mixtures M19 to M26.

Comparison between the experimentally measured 28-day compressive strength and the estimated values as obtained from each of the developed models are presented in Figure 9, Figure 10 and Figure 11. The actual values of the estimated compressive strength for each of the developed 28-day compressive strength model are provided in Appendix B. In mixtures M1 to M9, presented in Figure 9, the results of each of the model are very close to the experimental values, except the estimates of the hybrid GA-SVR-CSD for mixture M6 to M9 that show slight deviation from the measured values. For mixtures M10 to M18, presented in Figure 10, the results of each of the developed hybrid models agree excellently well with the measured values, except the results of hybrid GA-SVR-CS28A for mixtures M24 to M26 as shown in Figure 11. In all, the results of each of the models are excellent, while some models perform better than the others.

### 3.5. Modeling the Influence of Constituents of AALNM on the Compressive Strength Using the Developed Hybrid GA-SVR-CS28A Model

**Figure 12:**
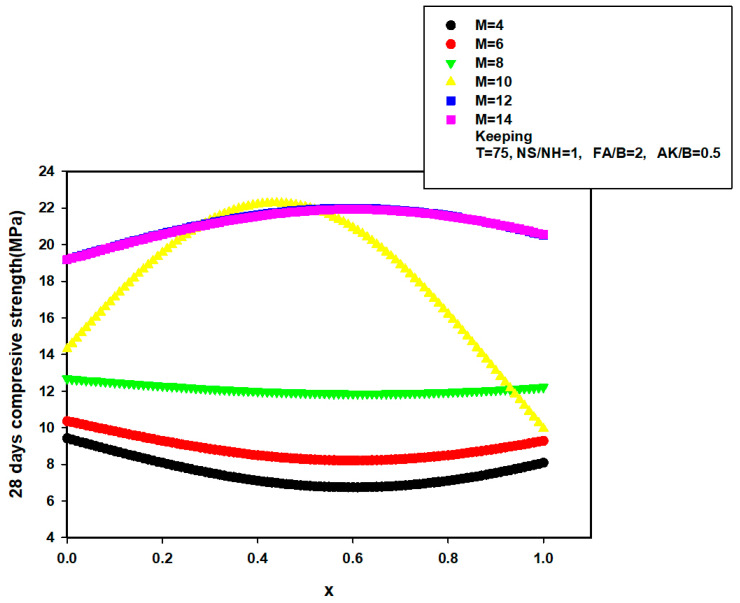
Dependence of 28-day compressive strength of AANLM at various molarity on the binder ratio using the developed hybrid GA-SVR-CS28A model.

**Figure 13:**
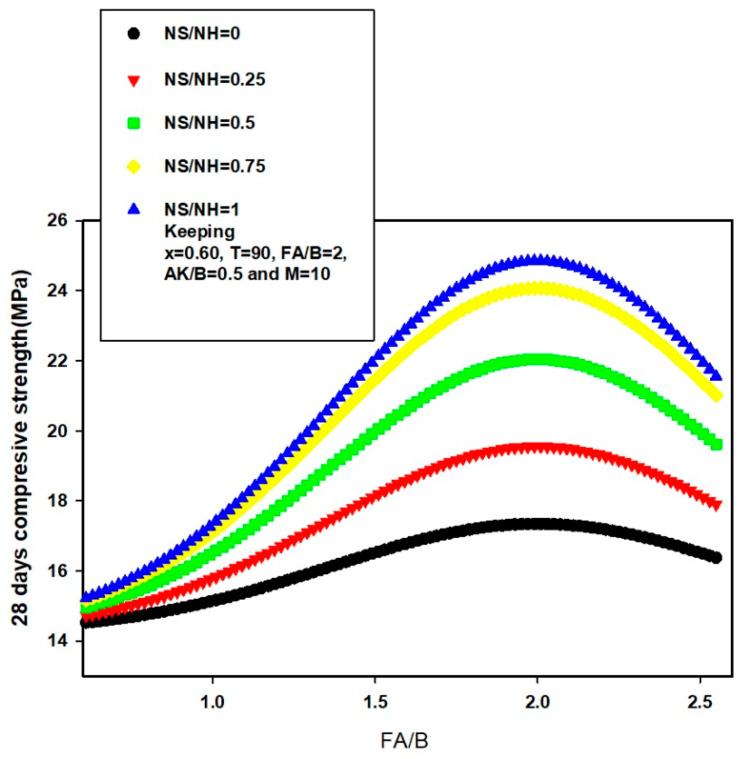
Dependence of 28-day compressive strength of AANLM at various molarity NS/NH ratio on the ratio of FA/B using the developed hybrid GA-SVR-CS28A model.

In order to further validate and justify the reported potentials and excellence of the developed 28-day compressive strength model, the influence of binary binder ratio on the 28-day strength of AALNM was investigated at different molarity of sodium hydroxide using the developed hybrid GA-SVR-CS28A. The results of investigation are presented in Figure 12, keeping the curing temperature (T), sodium silicate to sodium hydroxide (NS/NH) ratio, fine aggregate to binder ratio (FA/B), and alkaline to binder ratio (AK/B) at 75 °C, 1, 2, and 0.5, respectively. 28-days compressive strength decreases in a parabolic form characterized with minimum point as the ratio of binary binder increases for lower sodium hydroxide molarity of 4, 6, and 8. A sudden rise in 28-day strength was observed when the molarity of sodium hydroxide was 10M after which further increase in the molarity decreased the strength. It should be noted that the developed hybrid GA-SVR-CS28A model was only supplied with input descriptors while the model implements its acquired and saved support vectors during the training phase of model development for its estimation as presented in Figure 12. The result of similar investigation is revealed in Figure 13 where the dependence of the 28-day strength on fine aggregate to binder ratio at different sodium silicate to sodium hydroxide (NS/NH) ratio is conducted. The maximum 28-day achievable strength increases with an increase in NS/NH ratio and begins to decrease thereafter.

## 4. Conclusions

The compressive strength of alkali-activated natural pozzolan/limestone powder mortar (AALNM) is modelled through hybridization of support vector regression (SVR) and genetic algorithm (GA) using binary binder variation, sodium hydroxide molarity (NH), curing temperature, sodium silicate to sodium hydroxide (NS/NH), alkaline to binder ratio [AK/B], and fine aggregate to binder ratio (FA/BD) as descriptive features. The developed hybrid GA-SVR-CS1, GA-SVR-CS3, and GA-SVR-CS14 models are capable of estimating one-day strength, three-day strength, seven-day strength, and 14-day strength of AALNM, respectively, with accuracy of 96.64%, 90.84%, and 93.4% measured on the basis of correlation coefficient between the estimated strength and experimentally measured values. The developed 28-day strength models include the hybrid GA-SVR-CS28A model with six descriptive features, GA-SVR-CS28B model that uses one-day strength, GA-SVR-CS28C model that employs three-day strength, GA-SVR-CS28D model that implements seven-day strength and GA-SVR-CS28E that estimates 28-day strength using 14-day strength as its input. Each of the developed 28-day strength is characterized with excellent predictive and generalization strength. The developed hybrid models were also employed to model the behavior of AALNM and the obtained behavior agree perfectly well with the experimental data. The hybrid models proposed and developed in this research are meritorious because of their strengths, accuracy, and precision, coupled with their ability to save appreciable experimental time and other valuable resources.

## Figures and Tables

**Table 1:** Mixture proportions of alkali-activated natural pozzolan/limestone powder mortar specimens.

Mix	X -	M mol/dm^3^	T (°C)	NS/NH -	FA/B -	AK/B -	CS-1 (MPa)	CS-3 (MPa)	CS-7 (MPa)	CS-14 (MPa)	CS-28 (MPa)
M1	0	10	75	1	2	0.5	4.4	4.9	5.2	7.4	8.9
M2	0.2	10	75	1	2	0.5	18.6	18.7	21.9	22.4	22.6
M3	0.4	10	75	1	2	0.5	20.7	23.9	24.3	24.5	25.0
M4	0.6	10	75	1	2	0.5	20.9	24.0	25.2	25.3	27.0
M5	0.8	10	75	1	2	0.5	12.9	14.0	14.5	15.3	15.7
M6	1	10	75	1	2	0.5	5.3	5.6	5.8	6.0	6.3
M7	0.6	4	75	1	2	0.5	4.3	4.4	4.8	5.3	6.0
M8	0.6	6	75	1	2	0.5	4.7	4.9	5.0	5.6	7.6
M9	0.6	8	75	1	2	0.5	7.9	8.4	9.0	9.8	11.6
M10	0.6	12	75	1	2	0.5	20.2	24.3	24.7	24.7	22.8
M11	0.6	14	75	1	2	0.5	21.6	23.6	24.7	24.0	22.7
M12	0.6	10	25	1	2	0.5	6.85	7.73	10.55	11.68	13.00
M13	0.6	10	45	1	2	0.5	10.68	12.47	12.76	13.25	14.13
M14	0.6	10	60	1	2	0.5	17.06	19.34	20.36	20.86	22.00
M15	0.6	10	90	1	2	0.5	23.35	24.13	24.37	24.84	25.92
M16	0.6	10	75	0	2	0.5	9.6	14.4	13.2	14.3	15.6
M17	0.6	10	75	0.5	2	0.5	15.4	18.3	18.9	19.1	18.9
M18	0.6	10	75	0.75	2	0.5	17.1	19.1	19.0	19.5	20.4
M19	0.6	10	75	1.25	2	0.5	18.3	18.9	18.8	15.3	16.2
M20	0.6	10	75	1.5	2	0.5	10.9	12.3	13.4	10.5	10.9
M21	0.6	10	75	1	1.4	0.5	15.4	18.3	20.1	22	23.5
M22	0.6	10	75	1	1.6	0.5	17.6	20.6	22.4	23.3	24.3
M23	0.6	10	75	1	1.8	0.5	18.2	21.2	23.6	24.2	25
M24	0.6	10	75	1	2.2	0.5	19.5	21.3	22.8	23.4	24.4
M25	0.6	10	75	1	2	0.45	20.3	22.2	23.7	25.0	23.7
M26	0.6	10	75	1	2	0.55	21.5	23.6	22.8	23.1	22.5

**Table 2:** Outcomes of the statistical analysis performed on the dataset used for modelling and simulation.

Parameter	Mean	Range	Standard Deviation	CC-1 (MPa)	CC-3 (MPa)	CC-7 (MPa)	CC-14 (MPa)	CC-28 (MPa)
X	0.577	1	0.173	−0.026	−0.007	−0.035	−0.073	−0.095
M(mol/dm^3^)	9.769	10	1.728	0.578	0.605	0.609	0.589	0.545
T (°C)	71.923	65	12.006	0.329	0.308	0.255	0.236	0.226
NS/NH	0.962	1.5	0.252	0.083	−0.021	0.032	−0.042	−0.055
FA/B	1.962	0.8	0.150	−0.055	−0.102	−0.144	−0.189	−0.225
AK/B	0.500	0.1	0.014	0.027	0.029	−0.016	−0.039	−0.024
CS-1 (MPa)	14.737	19.05	6.187					
CS-3 (MPa)	16.556	19.9	6.871					
CS-7 (MPa)	17.376	20.4	7.053					
CS-14(MPa)	17.714	20	7.004					
CS-28 (MPa)	18.328	21	6.655					

**Table 3:** Optimized hyper-parameters of the early-age strength compressive strength models.

Model	Regularization Factor	Epsilon	Kernel Option	Hyper-Parameter Lambda	Kernel Function
Hybrid GA-SVR-CS1	418.5247	0.7126	0.3201	0.1	Gaussian
Hybrid GA-SVR-CS3	417.1485	0.002	0.4056	0.1	Gaussian
Hybrid GA-SVR-CS7	36.7688	0.0539	2.0000	0.1	Gaussian
Hybrid GA-SVR-CS14	457.0419	0.002	0.2433	0.1	Gaussian

**Table 4:** Reliability measurement for the early-age and 14-day compressive strength models.

Model	Training	Testing	Validation
Hybrid GA-SVR-CS1			
CC	0.9315	0.9779	0.9664
RMSE	2.1655	2.7192	1.8432
MAE	1.5296	2.4756	1.4143
Hybrid GA-SVR-CS3			
CC	0.9952	0.6755	0.9084
RMSE	1.1651	3.7007	1.4896
MAE	0.9163	2.5532	1.2969
Hybrid GA-SVR-CS7			
CC	0.7039	0.8284	0.6557
RMSE	5.2789	4.6159	5.0503
MAE	3.108	4.0925	4.9916
Hybrid GA-SVR-CS14			
CC	0.9768	0.9541	0.934
RMSE	2.1681	2.7784	3.4103
MAE	1.5633	2.3586	2.7793

**Table 5:** Optimized hyper-parameters of 28-days compressive strength models.

Model	Regularization Factor	Epsilon	Kernel Option	Hyper-Parameter Lambda	Kernel Function
Hybrid GA-SVR-CS28A	689.1925	0.002	0.6379	0.1	Gaussian
Hybrid GA-SVR-CS28B	15.124	0.5571	2	0.1	Gaussian
Hybrid GA-SVR-CS28C	7.7528	0.3432	2	0.1	Gaussian
Hybrid GA-SVR-CS28D	1.000	0.002	2	0.1	Gaussian
Hybrid GA-SVR-CS28E	13.7523	0.002	2	0.1	Gaussian

**Table 6:** Performance measuring parameters for the developed 28-days compressive strength model.

Model	Training	Testing	Validation
Hybrid GA-SVR-CS28A			
CC	0.9684	0.9899	0.6548
RMSE	2.2452	0.8442	3.8911
MAE	1.7507	0.6908	3.1246
Hybrid GA-SVR-CS28B			
CC	0.9315	0.9779	0.9664
RMSE	2.1655	2.7192	1.8432
MAE	1.5296	2.4756	1.4143
Hybrid GA-SVR-CS28C			
CC	0.9502	0.9958	0.9288
RMSE	1.9366	1.442	2.669
MAE	1.4209	1.1161	2.4128
Hybrid GA-SVR-CS28D			
CC	0.9392	0.9539	0.9675
RMSE	4.1867	1.0493	3.1514
MAE	3.087	0.8511	2.7583
Hybrid GA-SVR-CS28E			
CC	0.9947	0.9945	0.976
RMSE	0.7669	1.0394	1.4688
MAE	0.5998	0.8671	1.226

## Data Availability

The raw data required to reproduce these findings are available in the cited references in Section 2.3 of this manuscript.

## Appendix A

**Table A1:** Experimental and actual values of estimated early-day compressive strength models.

Mix	Experimentally Measured CS 1 (MPa)	Hybrid GA-SVR CS1 (Mpa)	Experimentally Measured CS3 (MPa)	Hybrid GA-SVR CS3 (Mpa)	Experimentally Measured CS-14 (MPa)	Hybrid GA-SVR- CS14 (MPa)
M1	4.430	7.351	4.850	5.600	5.200	19.904
M2	18.620	15.926	18.680	16.770	21.850	19.639
M3	20.720	21.579	23.890	23.309	24.300	19.292
M4	20.900	19.498	23.950	22.378	25.180	18.870
M5	12.870	12.546	13.980	15.079	14.500	18.387
M6	5.330	6.980	5.570	7.253	5.800	17.857
M7	4.320	5.726	4.430	5.218	4.790	11.022
M8	4.730	6.098	4.850	5.600	5.030	5.935
M9	7.850	12.657	8.360	8.791	9.010	9.533
M10	20.150	18.821	24.310	23.287	24.730	24.361
M11	21.550	20.094	23.560	22.605	24.670	24.116
M12	6.850	8.026	7.730	13.080	10.550	16.778
M13	10.680	12.657	12.470	13.080	12.760	13.174
M14	17.060	16.012	19.340	13.080	20.360	16.778
M15	23.350	21.730	24.130	23.124	24.370	23.631
M16	9.580	10.530	14.430	14.350	13.170	18.625
M17	15.380	14.600	18.320	18.326	18.860	18.806
M18	17.060	16.012	19.130	21.657	19.040	18.854
M19	18.320	17.608	18.920	18.559	18.800	18.854
M20	10.900	14.628	12.340	13.097	13.410	18.806
M21	15.400	13.837	18.250	17.926	20.100	20.680
M22	17.600	15.790	20.640	20.383	22.400	20.121
M23	18.200	18.285	21.220	22.183	23.600	19.512
M24	19.500	18.285	21.320	20.779	22.800	18.216
M25	20.300	19.415	22.180	22.146	23.650	18.870
M26	21.500	19.415	23.600	22.473	22.840	18.870

## Appendix B

**Table A2:** Experimental and actual values of estimated 28-day compressive strength models.

Mix	Experimentally Measured CS 28 (MPa)	Hybrid GA-SVR CS-28A (MPa)	Hybrid GA-SVR CS-28B (MPa)	Hybrid GA-SVR CS-28C (MPa)	Hybrid GA-SVR CS-28D (MPa)	Hybrid GA-SVR CS-28E (MPa)
M1	8.920	14.300	8.363	8.577	14.540	8.871
M2	22.600	19.558	23.197	21.550	22.521	22.898
M3	25.000	22.197	22.931	23.374	23.464	24.961
M4	27.000	20.925	23.547	23.341	21.942	25.623
M5	15.700	16.191	17.124	15.981	16.163	16.068
M6	6.300	9.947	8.162	9.041	14.517	6.970
M7	6.000	6.746	8.540	8.631	14.666	7.040
M8	7.610	8.207	8.040	8.577	14.581	6.890
M9	11.620	11.860	14.673	12.284	15.252	11.983
M10	22.750	21.983	23.580	23.104	22.543	25.191
M11	22.690	21.924	23.648	23.533	22.615	24.321
M12	13.000	14.230	12.023	11.693	15.253	13.941
M13	14.130	14.230	15.040	14.663	15.505	15.190
M14	22.000	21.292	22.107	22.066	20.217	22.007
M15	25.920	24.855	24.499	23.231	22.946	25.333
M16	15.570	15.819	15.439	16.581	15.621	15.642
M17	18.880	20.433	20.756	21.316	18.193	19.757
M18	20.420	21.869	22.107	21.887	18.380	20.286
M19	16.170	17.439	23.011	21.722	18.134	16.082
M20	10.900	12.845	15.056	14.595	15.702	12.743
M21	23.500	23.453	20.776	21.272	19.805	22.719
M22	24.300	23.693	22.501	23.294	23.118	23.515
M23	25.000	22.197	22.931	23.374	23.464	24.961
M24	24.400	18.649	23.541	23.733	23.392	23.614
M25	23.650	21.063	23.573	23.884	23.447	25.492
M26	22.500	20.747	23.633	23.516	23.411	23.345
